# Regeneration, Plasticity, and Induced Molecular Programs in Adult Zebrafish Brain

**DOI:** 10.1155/2015/769763

**Published:** 2015-08-31

**Authors:** Mehmet Ilyas Cosacak, Christos Papadimitriou, Caghan Kizil

**Affiliations:** ^1^German Centre for Neurodegenerative Diseases (DZNE), The Helmholtz Association, Arnoldstraße 18, 01307 Dresden, Germany; ^2^DFG-Center for Regenerative Therapies Dresden (CRTD), Cluster of Excellence at the TU Dresden, Fetscherstraße 105, 01307 Dresden, Germany

## Abstract

Regenerative capacity of the brain is a variable trait within animals. Aquatic vertebrates such as zebrafish have widespread ability to renew their brains upon damage, while mammals have—if not none—very limited overall regenerative competence. Underlying cause of such a disparity is not fully evident; however, one of the reasons could be activation of peculiar molecular programs, which might have specific roles after injury or damage, by the organisms that regenerate. If this hypothesis is correct, then there must be genes and pathways that (a) are expressed only after injury or damage in tissues, (b) are biologically and functionally relevant to restoration of neural tissue, and (c) are not detected in regenerating organisms. Presence of such programs might circumvent the initial detrimental effects of the damage and subsequently set up the stage for tissue redevelopment to take place by modulating the plasticity of the neural stem/progenitor cells. Additionally, if transferable, those “molecular mechanisms of regeneration” could open up new avenues for regenerative therapies of humans in clinical settings. This review focuses on the recent studies addressing injury/damage-induced molecular programs in zebrafish brain, underscoring the possibility of the presence of genes that could be used as biomarkers of neural plasticity and regeneration.

## 1. Introduction

The brain is an intricate and complex network of hardwired neurons and glia that sustain a tremendously complex architectural integrity and central physiological function throughout the life of vertebrates. In contrast to what Ramon y Cajal proposed in 1928 [1], we now know that the nervous system is not fixed and immutable, but is quite plastic in its nature so as to respond to physiological and external stimuli. The terms of adult neurogenesis and plasticity, therefore, denote the overall ability of the brain—in general the nervous system—to remodel its cellular composition and synaptic wiring on demand.

## 2. Plasticity in Mammalian Brains Is Limited

The adult vertebrate brains display a large variety of neural plasticities, which includes the dynamic recruitment of the synapses, and neurogenesis upon the proliferative activity of the neural stem cells (NSCs). Neurogenesis in adult mammalian brain is a result of localized niches of stem cells [2–7]. In adult mammals, although several regions of the brains were suggested to be neurogenic [8–11], canonical zones are believed to exist in the telencephalon [12, 13] in two distinct neurogenic areas: the subventricular zone (SVZ) of the lateral ventricle and the subgranular zone of the dentate gyrus in the hippocampus (SGZ) [2, 3, 14–17]. In rodents, the SVZ niche consists of heterogeneous neural stem cells that give rise to different cell types [7, 18]. The SVZ contains relatively quiescent astrocyte-like neural stem cells and these astrocytes get activated upon damage or injury yielding in quite poor regeneration due to scarce newborn neurons, inability to form lost neuronal cell types, and low survival [19–27]. Another type of astrocytic cells is the parenchymal astroglia, which is one of the major cell types reacting to any injury by increasing their proliferation rate [24, 28–30]. Despite their neurogenic potential* in vitro*, these astroglia do not form neurons* in vivo* [28, 31–33]. Upon injury, parenchymal astrocytes remain within their lineage and amplify themselves as a scar is formed [28, 34–36]. Such a gliotic scar hampers axonal regeneration by generating an impermeable physical barrier [37–39], which exacerbates the insufficient cellular reconstitution and neural recuperation.

Several stimuli including traumatic injuries, chronic loss of neurons, environmental changes, cognitive input, and disease states can induce plasticity response in the brain [40–46]. The disruption of such a plasticity response and mutilation of adult neurogenesis not only are causes of improper regenerative ability, but also lead to cognitive impairment and psychiatric disorders [47, 48]. For instance, hippocampal atrophy and reduced adult neurogenesis due to impaired activity of the NSCs were found to correlate with the cognitive dysfunction and memory performance [49]. Additionally the fact that some antipsychotic drugs elevate the proliferation of the NSCs [50] suggests a strong functional relevance of adult neurogenesis to schizophrenia—the exact cause of which is unknown but the onset and progression of the disease correlate with wrongly structured or absent neural circuits involved in production of neurotransmitters such as dopamine or the ones associated with cognitive functions. One hallmark of the pathophysiology of the psychiatric disorders is reduced size of the hippocampus—a prominent region of the brain involved in formation of memory, spatial navigation, and consolidation of thought. Since hippocampus is a region that generates neurons throughout the lifespan of humans utilizing neural stem cells, such observations suggest that the reduced plasticity of neural stem cells (NSCs) and hampered adult neurogenesis might be a major cause of psychiatric disorders.

Severe neuronal damage in case of medial cerebral arterial occlusion (MCAO) or ischemic injury was also shown to induce plasticity in mammalian brains [19, 27, 51–53]. MCAO results in infarcts and neuronal death in large regions of the brain including the striatum and cortex. Upon such an insult, the progeny of the NSCs at the SVZ diverts their normal migratory routes to these nonneurogenic regions and generates neurons that populate the infarct areas [19, 27]. Although the number of neurons is meager, the subtypes of the neurons are not exactly matching the lost ones and the survival of newborn neurons is poor. Additionally, mammalian brains were also suggested to bear plasticity upon neurodegenerative conditions [41, 43, 45, 54], although this ability is not fully translated into functional recovery. Several studies have shown that neural stem cell is affected during chronic neurodegeneration; for instance, postmortem analyses of Huntington's patients showed thicker SVZ and increased proliferation of ventricular cells [41], chemically induced epileptic seizures transiently increase the production of neuroblasts in the hippocampus and the SVZ [45], and, in an experimental model of murine prion disease and postmortem analyses of Creutzfeldt-Jacob patients, hippocampal neurogenesis was found to increase [42], while in Parkinson's patients cell proliferation is dramatically hampered [43]. These findings constitute an overall indication that mammalian brains might have a widespread but unfavorable plasticity response, which endows us an incentive for aiming at regenerative therapies by manipulating the stem cell behavior* in vivo*.

## 3. Zebrafish Has an Extensive Plasticity in Its Adult Brain

In nature, in contrast to mammals, several vertebrates display a striking ability of widespread adult neurogenesis and brain plasticity [5, 55–58]. One of these organisms is zebrafish, which possess an extensive adult neurogenesis response of its NSCs and can regenerate its brain upon traumatic lesions [59–62]. This is in stark contrast to mammalian brains, which poorly regenerate, despite prevalent adult neurogenesis in two neurogenic niches of the forebrain. Various zones of stem cell activity were described in adult zebrafish brain [63–67]. These zones generate neurons that are integrated into the circuitry as BrdU labeling experiments resulted in various lineages of newborn neurons in parenchymal regions after several weeks of BrdU pulse [63, 64]. The majority of the stem/progenitor cells are of radial glial cells (RGCs) [5, 56, 68]. RGCs express markers such as GFAP, glutamine synthetase, vimentin, S100B, aromatase-B, BLBP, or* her4.1* [60, 63, 64, 69–72]. With such properties, adult zebrafish brain is quite more plastic than their mammalian counterparts. Additionally, in contrast to mammals, the adult fish brain regenerates even after severe traumatic lesions without overt scar formation [60, 61]. Injury to the dorsal telencephalon elevates the levels of the proliferation of ventricularly located neurogenic progenitors: RGCs [59, 60, 62]. Thus, mammals and zebrafish have a substantial difference in their abilities to recuperate neuronal damage in their central nervous system. Additionally, the genes and pathways involved in the initiation and maintenance of such an extensive regenerative response in zebrafish brain are largely unknown, rendering zebrafish as an excellent model to investigate those molecular programs.

## 4. Induced Molecular Programs Enable Plasticity Response during Zebrafish Brain Regeneration

**Figure 1:**
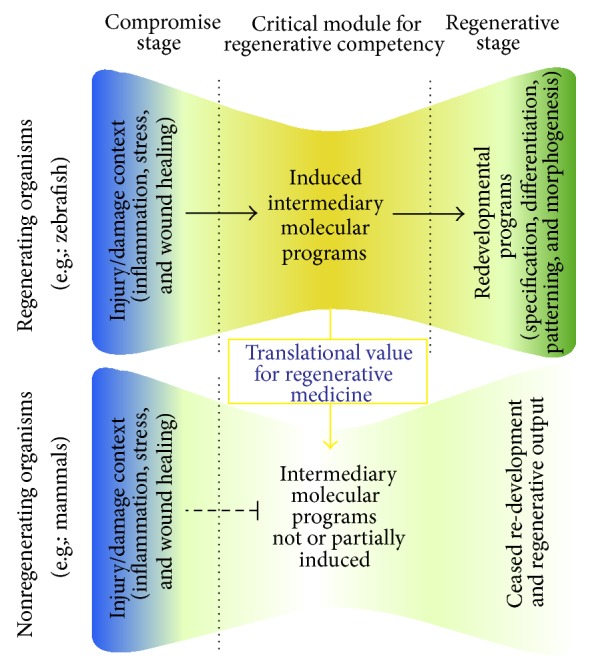
Induced intermediary molecular programs enable regeneration. In regenerating and nonregenerating organisms, injury or damage leads to similar initial events such as inflammation, stress, and wound healing response. There is increasing evidence that regenerating organisms such as zebrafish induce the expression of genes that are functionally essential for regenerative response including the modulation of stem cell plasticity, cell proliferation, differentiation, and survival. These genes and pathways constitute the “induced intermediary molecular programs,” which set up the stage for reopening the developmental programs of specification, differentiation, patterning, and morphogenesis. One of the reasons why regeneration is not efficient in mammals could be the lack of activation of these intermediary genes. Therefore, the intermediary molecular programs bear a significant value for translational aspects of regenerative medicine and can be used as biomarkers of plasticity and regenerative ability.

The process of regeneration definitely involves turning on “redevelopment.” For instance, if a neuron will be generated, genes that govern the specification and differentiation of that particular subtype of neuron during development—such as Delta-Notch signaling or pathways leading to subtype specification, axonogenesis, or synaptogenesis—become active again. However, in case of neuronal loss, be it acute or chronic, nonphysiological events that are normally not seen during development take place. These include stress response, inflammation, wound healing mechanisms, and other phenomena related to the breach of the homeostatic balance. In most cases, these phenomena were shown to be detrimental for the regenerative ability in mammals [73–80], and they have to be overcome for regeneration to succeed. On the other hand, zebrafish can regenerate even though experiencing such nonphysiological circumstances. Therefore, a plausible hypothesis is that the organisms that can regenerate might be using some “intermediary” molecular programs that link the initial events to the redevelopment of tissues (Figure 1). These intermediate programs could be specifically induced after neuronal loss and might be crucial to regenerative success as they might set the stage to alleviate the negative consequences of homeostatic compromise and to turn on the programs of redevelopment. A scientific challenge based on this hypothesis is to identify such putative intermediary genes and pathways in regenerating organisms. Thus, zebrafish serves as a promising animal model to this purpose.

Several studies have so far shown that, during regeneration of the adult zebrafish tissues, genes that are not expressed during the development of the corresponding tissues can be induced [81–91]. Specifically in adult zebrafish brain, acute inflammation has been shown to contribute to activation of neural progenitor cells with radial glial identity [77, 86]. Leukotriene C4 (LTC4) was shown to emanate from immune cells that populate the brain tissue after lesion and activate an intracellular signal transduction in radial glial cells, where the cysteinyl leukotriene receptor 1 (*cystlr1*) is present [86]. Injection of LTC4 using cerebroventricular microinjection (CVMI) [70, 92] is sufficient to increase the proliferation of radial glial cells and subsequent regenerative neurogenesis by activating regeneration-specific molecular program involving the zinc finger transcription factor* gata3*. This gene is interesting as it is not expressed during development and homeostatic adult telencephalons of the zebrafish brain, but is induced in the RGCs shortly after lesion [84]. Knockdown experiments using CVMI and Gata3 antisense morpholinos showed that Gata3 does not partake in regulation of constitutive neurogenesis, but is specifically required for the injury-induced cell proliferation response of the ventricular neurogenic progenitor cells and subsequent reactive neurogenesis: two hallmarks of the regenerative response gata3 are injury induced in other regenerating organs of zebrafish and are functionally required for the proliferation of progenitor cells [84]. Such a dynamic expression and biological relevance of gata3 suggests that this gene might be part of a molecular program zebrafish might be using universally for regenerating its tissues. Additionally,* gata3* has not been documented to be activated in mammalian brains upon injury or insult so far, suggesting that such genes like* gata3* might underlie the disparity between the regenerative capacities of zebrafish and mammalian brains. Therefore, such molecular programs or novel epistatic interactions could be used as biomarkers of brain injury and regenerative response.

Another study identified the 7-pass transmembrane domain chemokine receptor* Cxcr5* as a gene required for regenerative neurogenesis but not for increased proliferation of the radial glial cells [83]. Cxcr5 is expressed at low levels in the RGCs in homeostatic unlesioned adult zebrafish telencephalon and is predominantly absent in neurons. After a lesion,* cxcr5* expression increases dramatically in periventricular neurons [83]. Blocking this chemokine signaling by overexpressing a dominant negative version of the Cxcr5 receptor that lacks the transmembrane domains 5, 6, and 7, which renders the receptor incapable of eliciting an intracellular signaling cascade, does not result in any change in RGC proliferation in unlesioned or lesioned brains. However, the same genetic knockdown results in reduced number of newborn neurons only after lesion [83]. Similarly, morpholino-mediated knockdown of* cxcr5* gene in adult zebrafish brain leads to similar reduction of regenerative neurogenesis [83]. Conversely, when the full-length Cxcr5 is overexpressed, production of new neurons increased significantly only after lesion despite no change in RGC proliferation. These findings suggest that Cxcr5-mediated chemokine signaling might be specifically required for generation of neurons after acute neuronal loss and might also serve as a biomarker for regenerative neurogenesis.

Alternatively, some molecular programs could be turned off or overridden during regeneration of adult zebrafish brain [93]. For instance, estradiol was shown to hamper proliferation of progenitor cells in the adult zebrafish brain under homeostatic conditions, while this regulation does not take place during regeneration [93]. Since radial glial cells specifically express the aromatase that synthesizes estrogen [72], certain physiological conditions might downregulate signaling pathways that are prevalent during homeostatic state.

Collectively, an important but still partial list of molecular programs that allow the special regenerative response in the zebrafish brain was identified as described above. Interestingly, some of those programs are induced only during regenerative stage and are essential for production of newborn neurons. These findings suggest that regenerating organisms such as zebrafish could use special molecular programs to enable regenerative neurogenesis, and these programs might be responsible for different regenerative capacities of zebrafish and mammalian brains.

## 5. Missing Regeneration Programs in Mammals?

**Figure 2:**
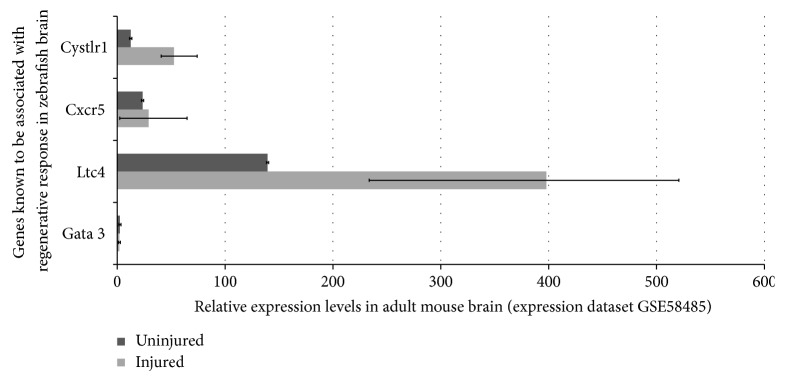
Relative expression levels of regeneration-associated genes of adult zebrafish brain in mouse brain before and after lesion based on publicly available gene expression datasets.

Experimental data suggests that the crucial need for induced intermediary programs in zebrafish makes regeneration possible [56, 77, 83, 84, 86]. A very valid and intriguing question is therefore whether those regeneration programs would be activated in mammalian brains after neuronal loss. Several gene expression datasets on central nervous system injuries are publicly available on repositories such as Array Express (http://www.ebi.ac.uk/arrayexpress/) and Gene Expression Omnibus (GEO) (http://www.ncbi.nlm.nih.gov/geo/). To find out whether the regeneration programs of zebrafish are activated in mammalian models of injury, we investigated the expression levels of those genes in one representative dataset (Figure 2). In this dataset, gene expression profiles of injured and uninjured mouse brains are compared. The expression values of the genes presented in the pathway analysis map are based on the Query Data Set GSE58484 (Gene Expression Omnibus accession number) [94]. The injured group reflects the gene expression from wild type B6 mice at 3 days after a traumatic brain injury at the ipsilateral neocortex (Datasets: GSM1412408, GSM1412409, and GSM1412410). The control samples show the gene expression values at the neocortex of uninjured wild type B6 mice (Datasets: GSM1412411, GSM1412412, and GSM1412413). When we checked the expression levels of three genes experimentally known to be required for regeneration in zebrafish,* gata3*,* cxcr5*,* cystlr1*, we found that* cystlr1* is expressed in high levels after injury, while* cxcr5* and* gata3* are unchanged (Figure 2). Interestingly,* gata3* expression is very low before and after injury, almost at nonexistent levels.* Cxcr5* is also expressed at low levels and statistically is not different than that of nondetectable levels. These findings suggest that it is quite possible that the inability to activate regeneration programs and genes, two of which are* cxcr5* and* gata3*, might be one of the underlying reasons why mammalian brains could not turn on regeneration mechanisms. This hypothesis also points to the importance of further experiments to elucidate more genes participating in regeneration response of adult zebrafish central nervous system.

**Figure 3:**
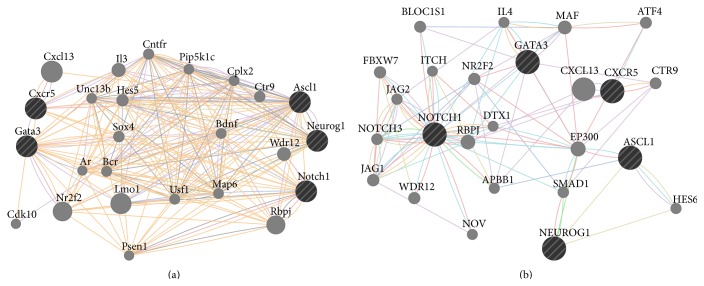
GeneMANIA interaction maps. Predicted interaction maps of* Gata3* and* Cxcr5* in mouse (a) and humans (b). See text for more details. Connections: red: physical interaction; violet: coexpression; orange: predicted; cyan: common pathway; blue: colocalization.

**Figure 4:**
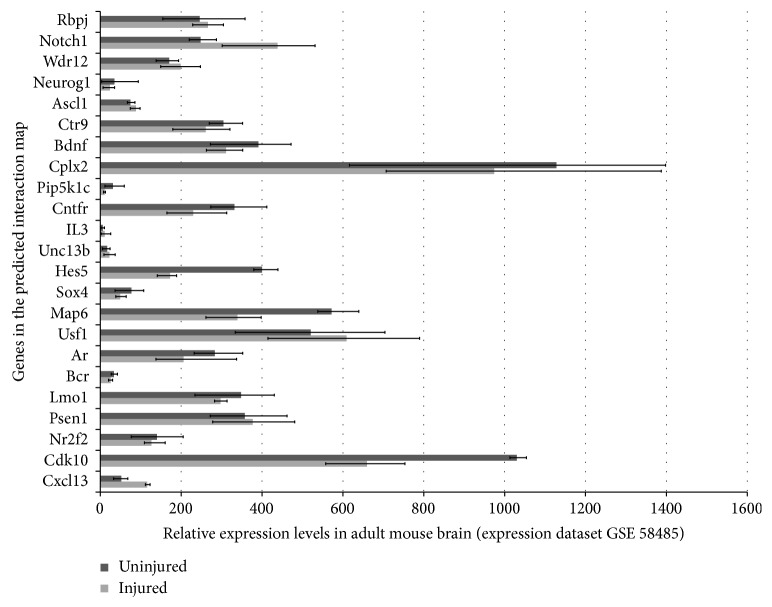
Relative expression levels of GeneMANIA-predicted map partners of Gata3 and Cxcr5 in experimental mouse brain injury gene expression datasets. See text for details.

**Figure 5:**
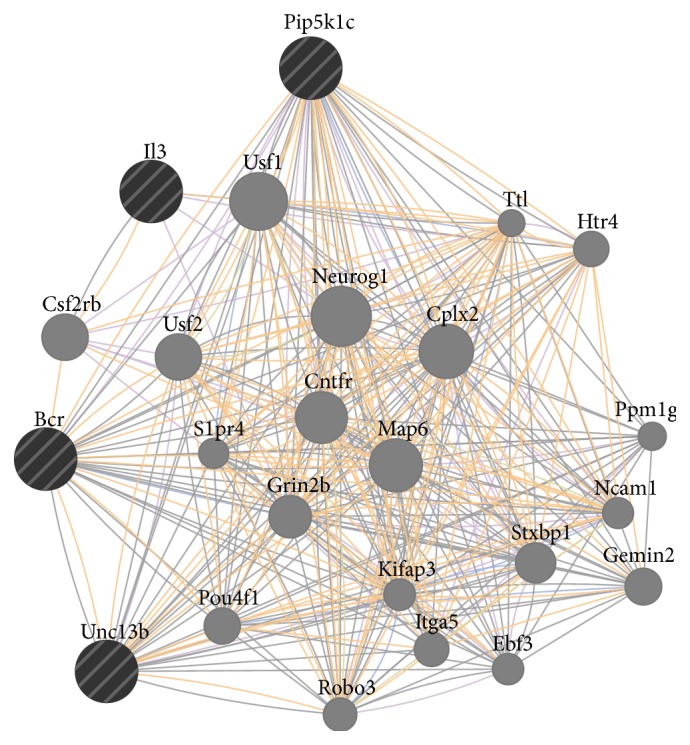
GeneMANIA iteratively predicted interaction map. The interaction map of genes that might be regulated by regeneration factors based on experimental injury models of mouse brain.* Il3*,* Pip5k1c*,* Bcr*, and* Unc13b* generate a map that contains genes that could be regulated by these genes if they would be expressed or regulated by regeneration factors in mouse brain. The map partners include various genes related to neurogenesis, such as* Neurog1*,* Robo3*, and* Pou4f1*.

Hypothetically, the regeneration genes if turned on in mammalian brains could modulate further pathways and genes. This modulation might run through two ways: (1) regeneration genes can regulate downstream genes and pathways that are already known to be associated with them; (2) regeneration genes could regulate completely novel genes and pathways. The latter scenario is impossible to predict without experimental studies, which will aim to identify downstream gene regulation of regeneration factors after misexpression studies such as knockdown, knockout, or overexpression. However, the former scenario can be predicted using already existing interaction maps. In order to find out this interaction map and pathway analysis for* gata3* and* cxcr5*, we used publicly available online in silico tools, such as GeneMANIA, a prediction tool for functional interaction maps and pathways based on a large data of functional interaction data (http://genemania.org/). When we included* gata3* and* cxcr5* into query and also added three proneural genes Neurogenin1 (Ngn1), achaete-scute complex homolog 1 (Ascl1), and Notch1 to narrow down the interaction map to neurogenic pathways, we found two particular maps for human and mouse (Figure 3). These maps revealed several potential map partners, which are hypothetically the genes that could be regulated if* gata3* and* cxcr5* would have been expressed in mammalian brains after injury regarding the first scenario above. When we analyzed the expression levels of those potential map partners in the original mouse brain injury dataset, we found that several of these genes—for instance,* Bcr*,* Unc13b*,* IL3*, and* Pip5k1c*—were either expressed at very low levels or unexpressed (Figure 4). These genes are taking part in regulating diverse events including cell cycle, neurotransmitter release, long-term potentiation of synapses, second messenger pathways, cytokine signaling, cell fate determination, and cell migration [95–102]. Thus, activation of regeneration factors in mammals could have the potential to modulate all these molecular events, which might be misregulated in the absence of such factors, two of which could be* gata3* and* cxcr5*. As new molecular players will be identified experimentally, the interaction and regulation map could be widened. Potential candidates could also be analyzed for their expression and function in mammalian central nervous system to see whether they could convey the regenerative ability in mammalian nervous tissue. For instance, when predicted interaction maps and pathway analysis are made for* Bcr*,* Unc13b*,* IL3*, and* Pip5k1c* in mouse, several genes are included in the map (Figure 5), which could serve as a starting point for functional epistatic analyses of regenerative ability.

## 6. Neurodegeneration as a Means of Addressing Stem Cell Plasticity in Zebrafish

Specific regions of the zebrafish brain are strikingly conserved with mammalian brains [103, 104], and this allows zebrafish to be used as an excellent model for neurodegeneration. Several transgenic or mutant zebrafish lines were generated to model neurodegeneration in fish [105–108]. Various techniques ranging from morpholino knockdown of specific disease-related genes [109, 110] to the use of neuronal promoters for driving mutant versions of different neurodegeneration-associated proteins [111–116], or generating mutants for loss of/function studies [117–122] were used. These models provide important information of the pathophysiology of the disease progression and underlying molecular programs. Like the genes induced after traumatic injury in zebrafish brains, neurodegeneration models are also likely to give us insights on biomarkers that could be pragmatically utilized in regenerative medicine. These biomarkers can also be used to find out the “druggable” candidates that could be harnessed in clinical settings for regenerative therapies.

## 7. Conclusion

Zebrafish serves as a yet developing but quite promising organism for modeling human diseases [123]. The premise of zebrafish is its ease in getting at the mechanisms underlying the* in vivo* regenerative aptitude. Understanding such mechanisms would thus be instrumental in addressing questions on the presence of special molecular mechanisms and on whether we can activate those programs in mammalian brains to achieve functional recovery utilizing the endogenous stem cells. Here, we wish to underscore the need to further test the hypothesis that induced molecular programs utilized by adult zebrafish brain might give insight into how we can coax mammalian neural stem cells to proliferate and enhance the adult neurogenesis response in compromised adult brains. With simple* in silico* tools, such genes and pathways can also help researchers to hypothesize the molecular basis of regenerative ability also in mammalian brains.
